# Ferritin and its association with anaemia in a healthy adult population in Kenya

**DOI:** 10.1371/journal.pone.0275098

**Published:** 2022-10-14

**Authors:** Geoffrey Omuse, Assumpta Chege, David Enoch Kawalya, Elizabeth Kagotho, Daniel Maina

**Affiliations:** Department of Pathology, Aga Khan University Hospital, Nairobi, Kenya; Stellenbosch University Faculty of Medicine and Health Sciences, SOUTH AFRICA

## Abstract

**Background:**

Iron deficiency is the commonest cause of anaemia worldwide. Serum ferritin is the most sensitive non-invasive indicator of iron stores but its utility is compromised in inflammatory states as it is an acute phase reactant. This study sought to estimate the burden of iron deficiency in a healthy adult population residing in Kenya and to determine the association between various ferritin cut-offs and anaemia in a population known to have chronic low-grade inflammation.

**Methods:**

Healthy adults aged 18–65 years were recruited from urban towns in 4 counties in Kenya at average altitudes of 1683-2099m above sea level as part of a global study conducted by the International Federation of Clinical Chemistry (IFCC) to determine reference intervals (RIs) for common laboratory tests. We analyzed complete blood count (CBC), C-reactive protein, iron, transferrin, transferrin saturation and ferritin data.

**Results:**

We obtained data from 528 participants. There were 254 (48.1%) males and 274 females (51.9%). Based on a ferritin cut-off of 15 μg/L and Hb cut-offs of 14.5 g/dL and 12 g/dL, the prevalence of iron deficiency anaemia was 0.8% and 7.3% in males and females respectively. The odds of having anaemia was highest if one had a ferritin value less than 15 μg/L with a sensitivity of 28.6% and specificity of 98.4% in males, and sensitivity of 83.3% and specificity of 78.0% in females.

**Conclusion:**

Only the ferritin cut-off of 15 ug/L had an association with anaemia where it can be used for ruling out iron deficiency as the cause. Sex specific ferritin cut-offs for diagnosing iron deficiency in adults in sub-Saharan Africa need to be derived by comparing ferritin levels to stainable iron in bone marrow aspirates and trephines in order to ensure that we are using appropriate clinical decision limits.

## Introduction

Iron deficiency is the commonest cause of anaemia globally and is largely attributed to nutritional deficiencies especially in sub-Saharan Africa [1]. Functional iron deficiency can also occur secondary to iron sequestration in the reticuloendothelial system in patients with chronic inflammation such as chronic kidney disease (CKD), inflammatory bowel disease (IBD) and chronic heart failure (CHF) [2].

Diagnosis of iron deficiency anaemia (IDA) requires demonstration of low blood haemoglobin (Hb) in the presence of depleted iron stores [3]. Serum ferritin is the most sensitive non-invasive indicator of iron stores but its utility is compromised in inflammatory states as it is an acute phase reactant [3]. The presence of inflammation can reduce the accuracy of the World Health Organization (WHO) recommended serum ferritin cut-off of less than 15 μg/L used to diagnose iron deficiency in adults. WHO previously recommended a cut-off of 30 μg/L in settings with increased inflammation but this has since been revised to 70 μg/L [3,4]. Some guidelines have recommended a cut-off of 100 μg/L when diagnosing iron deficiency in patients with CKD or IBD [5,6].

Other biomarkers of iron status have inherent limitations. For instance, serum iron has a diurnal variation with levels being highest in the morning and has also been shown to reduce with an increase in body mass index (BMI) [7,8]. Serum transferrin saturation (TSat) is a useful marker especially in assessing iron overload but its calculation is dependent upon measurement of serum iron and transferrin levels; hence is influenced by factors affecting these two analytes. TSat has been proposed as a useful test in assessing iron deficiency in patients with chronic inflammatory states with a TSat below 20% indicative of iron deficiency [2]. Soluble transferrin receptor is a test that is able to distinguish between iron deficiency anaemia and anaemia secondary to chronic disease where it is usually increased in the former. However, the test is not readily available in sub-Saharan Africa and can be affected by conditions associated with erythroid hyperplasia.

Urbanization in sub-Saharan Africa has resulted in an increase in the prevalence of metabolic syndrome, a known cause of chronic low-grade inflammation [9]. The accurate assessment of iron deficiency in populations with chronic inflammation continues to be a challenge. The release of inflammatory cytokines such as interleukin 6 influences iron metabolism via the release of the iron regulatory hormone hepcidin which causes iron to be sequestered in the reticulo-endothelial system [10].

There is no consensus on how best to utilize markers of iron status such as ferritin and TSat in the diagnosis of iron deficiency especially in populations with inflammation [11]. The paucity of published data from sub-Saharan Africa (SSA) on reference intervals for markers of iron status further adds to concerns about the suitability of published cut-offs in assessment of iron deficiency in an African population. The presence of inflammation due to communicable and non-communicable diseases in people living in SSA could confound the utility of tests like ferritin hence the need to evaluate their diagnostic accuracy in diagnosing suspected iron deficiency anaemia.

This study sought to estimate the burden of iron deficiency in a healthy adult population residing in Kenya and to determine the association between ferritin cut-offs used to define iron deficiency and anaemia in a population known to have chronic low-grade inflammation due to metabolic syndrome whose prevalence has previously been reported as 25.6% [9,12]. Metabolic syndrome was determined using the 2009 harmonized criterion.

## Methods

We secondarily analyzed complete blood count (CBC), C- reactive protein (CRP), serum iron, transferrin, transferrin saturation and serum ferritin data collected as part of a global study conducted by the International Federation of Clinical Chemistry (IFCC) to determine RIs for common laboratory tests. Kenya was one of the participating countries and recruited participants using a harmonized protocol designed to standardize pre-analytical, analytical and post-analytical processes [13].

The study methodology and Kenyan RIs for complete blood counts, chemistry and immunoassays have previously been published [12,14]. In brief, healthy black Africans aged 18–65 years were recruited from urban towns in 4 counties in Kenya (Nairobi, Kiambu, Nakuru and Kisii) after obtaining informed written consent. The average altitude in metres above sea level of the counties is as follows: Nairobi– 1795m, Kisii– 1700m, Nakuru– 2099m and Kiambu– 1683m. Recruitment was stratified into 4 age groups: 18–29, 30–39, 40–49 and 50–65 years with a target of 60 males and females for each stratum. Exclusion criteria included a body mass index (BMI) >35 kg/m^2^, ethanol intake ≥70 g per day, smoking >20 cigarettes per day, on medication for a chronic non-communicable disease (diabetes mellitus, hypertension, hyperlipidemia, allergic disorders, depression), recovery from acute illness within the past 15 days, hospitalization due to surgery or injury, chronic infection (HIV, HBV or HCV, pregnant or within 1 year after delivery.

CBC was performed on a Beckman Coulter ACT 5 DIFF CP analyzer (Brea, California, US) at the PathCare laboratory based in Nairobi, Kenya. Chemistry tests were performed on serum using a Beckman Coulter AU 5800 (Brea, California, US) and immunoassays on the DXI (Brea, California, US) all done at the Path Care referral laboratory in Cape Town, South Africa.

The reference interval study was approved by the Aga Khan University Hospital Nairobi (2014/REC-46) and Stellenbosch University (S16/10/219) Health Research Ethics Committees. The study was conducted in conformity with the Declaration of Helsinki.

### Statistical analysis

Descriptive data was summarized as medians with corresponding interquartile ranges (IQRs). Comparison of medians between male and female participants was done using the Mann-Whitney U test. Chi square or Fisher’s exact test was used to determine the association between ferritin and TSat categories based on specific cut-offs. Odds ratios (ORs) for the presence of anaemia were also determined. Anaemia was defined using previously published reference interval lower limits for Hb from the same population. These were a Hb < 12.0 g/dL in females and < 14.5 g/dL in males [14]. A Spearman’s correlation was done to determine the association between ferritin and hsCRP. All analysis was done using IBM® SPSS® statistics version 23 (SPSS, Chicago, IL, USA). A p-value < 0.05 was considered statistically significant.

## Results

We analyzed data from 528 participants who had laboratory results for all the analytes of interest. There were 254 (48.1%) males and 274 females (51.9%). With the exception of red blood cell distribution width (RDW), there were statistically significant differences in Hb, red blood cell (RBC) parameters and markers of iron status between males and females. Both BMI and C-reactive protein (CRP) levels were higher in female participants. A summary of the participant characteristics is shown in **Table 1**.

**Table 1:** Descriptive characteristics.

	Male (n = 254)		Female (n = 274)		Comparison of medians (Male vs Female)
Variable	Median (IQR)	Min-Max	Median (IQR)	Min-Max	*p*-value
Age (years)	38 (19)	20–65	39 (20)	18–64	0.915
BMI (kg/m^2^)	24.87 (5.64)	16.29–34.94	26.08 (6.23)	17.10–38.05	0.029
Hb (g/dL)	16.7 (1.3)	8.2–19.4	14.2 (1.6)	7.2–18.8	0.000
MCV (fl)	88 (6)	57–97	86 (9)	53–99	0.005
MCH (pg)	30 (3)	17–34	29 (3)	16–33	0.000
MCHC (g/dl)	34 (1)	30–36	34 (1)	28–35	0.000
RBC (X10^12/L)	5.61 (0.56)	4.40–7.26	4.93 (0.53)	3.76–6.63	0.000
RDW	12.9 (1.1)	10.3–17.8	13.0 (1.6)	10.6–20.2	0.962
Ferritin (μg/L)	122 (142)	2–1297	29 (51)	2–483	0.000
Iron (μmol/L)	17.3 (6.9)	2.3–55.4	13.7 (8.5)	2.1–36.4	0.000
Transferrin (g/L)	2.4 (0.4)	1.5–4.4	2.7 (0.7)	1.8–4.4	0.000
TSat (%)	28 (12)	3–87	20 (14)	2–53	0.000
hsCRP (mg/L)	0.99 (2.00)	0.20–36.25	1.66 (3.14)	0.20–60.75	0.000

Key: BMI-Body Mass Index; Hb-Haemoglobin; hsCRP-highly sensitive C-reactive protein; MCH-Mean corpuscular haemoglobin; MCHC-Mean corpuscular haemoglobin concentration; MCV-Mean corpuscular volume; RDW-Red cell distribution width; TSat-Transferrin saturation.

Spearman’s correlation showed no association between ferritin and hsCRP levels (rs(526) = 0.059, *p* = 0.179).

Based on Hb cut-offs of 14.5 g/dL and 12 g/dL, 7 (2.6%) and 24 (9.4%) male and female participants had anaemia respectively giving an overall anaemia prevalence of 5.9%. Using WHO definitions of anaemia, only 1 male participant had a Hb < 13.0 g/dl giving an overall anaemia prevalence of 4.7%. Using a ferritin cut-off of <15 μg/L, the overall prevalence of iron deficiency anaemia was 4.0% (21 out of 528) using the WHO definition of anaemia and 4.2% (22 out of 528) using population specific Hb cut-offs. The association between anaemia defined using the population specific cut-offs and iron status based on various ferritin cut-offs is shown in **Table 2**. Based on a ferritin cut-off of 15 μg/L, the overall, male and female prevalence of iron deficiency was 15.3%, 2.4% and 27.4% respectively. The prevalence of iron deficiency anaemia based on the population specific Hb cut-offs and ferritin < 15 μg/L was 0.8% and 7.3% in males and females respectively. Using a ferritin cut-off of < 30 μg/L, the overall iron deficiency prevalence was 29.9%, while the prevalence in males and females was 7.1% and 51.1% respectively. The odds of having anaemia was highest if one had a ferritin value less than 15 μg/L with a sensitivity of 28.6% and specificity of 98.4% in males, sensitivity of 83.3% and specificity of 78.0% in females.

**Table 2:** Association between markers of iron status and anaemia.

		Anaemia											
		Male (Hb < 14.5 g/dl)				Female (Hb < 12.0 g/dl)				Male (Hb < 14.5) & Female (Hb < 12.0 g/dl)			
		Present	Absent	p-value	OR (95% CI)	Present	Absent	p-value	OR (95% CI)	Present	Absent	p-value	OR (95% CI)
		N = 7	N = 247			N = 24	N = 250			N = 31	N = 497		
Ferritin < 15 μg/L	Yes: n (%)	2 (28.6%)	4 (1.6%)	0.009	24.3(3.6–164.8)	20 (83.3%)	55 (22.0%)	0.000	17.7(5.8–54.0)	22 (71.0%)	59 (11.9%)	0.000	18.1(8.0–41.3)
	No: n (%)	5 (71.4%)	243 (98.4%)			4 (16.7%)	195 (78.0%)			9 (29.0%)	438 (88.1%)		
Ferritin < 30 μg/L	Yes: n (%)	3 (42.9%)	15 (6.1%)	0.009	11.6(2.4–56.6)	20 (83.3%)	120 (48.0%)	0.001	5.4(1.8–16.3)	23 (74.2%)	135 (27.2%)	0.000	7.7(3.4–17.7)
	No: n (%)	4 (57.1%)	232 (93.9%)			4 (16.7%)	130 (52.0%)			8 (25.8%)	362 (72.8%)		
Ferritin < 70 μg/L	Yes: n (%)	5 (71.4%)	67 (27.1%)	0.021	6.7(1.3–35.5)	23 (95.8%)	189 (75.6%)	0.024	7.4(1.0–56.1)	28 (90.3%)	256 (51.5%)	0.000	8.8(2.6–29.3)
	No: n (%)	2 (28.6%)	180 (72.9%)			1(4.2%)	61 (24.4%)			3 (9.7%)	241 (48.5%)		
Ferritin < 100 μg/L	Yes: n (%)	5 (71.4%)	91 (36.8%)	0.108	4.3(0.8–22.5)	23 (95.8%)	220 (88.0%)	0.247	3.1(0.4–24.1)	28 (90.3%)	311 (62.6%)	0.002	5.6(1.7–18.6)
	No: n (%)	2 (28.6%)	156 (63.2%)			1(4.2%)	30 (12.0%)			3 (9.7%)	186 (37.4%)		
TSat < 20%	Yes: n (%)	3 (42.9%)	38 (15.4%)	0.086	4.1(0.9–19.2)	21 (87.5%)	105 (42.0%)	0.000	9.7(2.8–33.3)	24 (77.4%)	143 (28.8%)	0.000	8.5(3.6–20.1)
	No: n (%)	4 (57.1%)	209 (84.6%)			3 (12.5%)	145 (58.0%)			7 (22.6%)	354 (71.2%)		

Key: CI-Confidence interval; Hb-Haemoglobin; OR-Odds ratio; TSat-Transferrin saturation.

All 22 participants with anaemia and a ferritin < 15 μg/L had a TSat < 20% as shown in **Table 3**. Out of 167 individuals with a TSat < 20%, only 67 (40.1%) had a ferritin < 15 μg/L while out of 81 individuals with a ferritin < 15 μg/L, 67 (82.7%) had a TSat < 20%.

**Table 3:** Association between transferrin saturation and ferritin at different cut-offs in assessing iron deficiency.

		Ferritin < 15 μg/L		Ferritin < 30 μg/L		Ferritin < 70 μg/L		Ferritin < 100 μg/L	
		Yes	No	Yes	No	Yes	No	Yes	No
**Anaemia**	Tsat < 20% (N = 24)	22 (91.7%)	2 (8.3%)	22 (91.7%)	2 (8.3%)	23 (95.8%)	1 (4.2%)	23 (95.8%)	1 (4.2%)
	Tsat 20% (N = 7)	0 (0.0%)	7 (100.0%)	1 (14.3%)	6 (85.7%)	5 (71.4%)	2 (28.6%)	5 (71.4%)	2 (28.6%)
**No anaemia**	Tsat < 20% (N = 143)	45 (31.5%)	98 (68.5%)	77 (53.8%)	66 (46.2%)	106 (74.1%)	37 (25.9%)	118 (82.5%)	25 (17.5%)
	Tsat 20% (N = 354)	14 (4.0%)	340 (96.0%)	58 (16.4%)	296 (83.6%)	150 (42.4%)	204 (57.6%)	193 (54.5%)	161 (45.5%)

**Fig 1:**
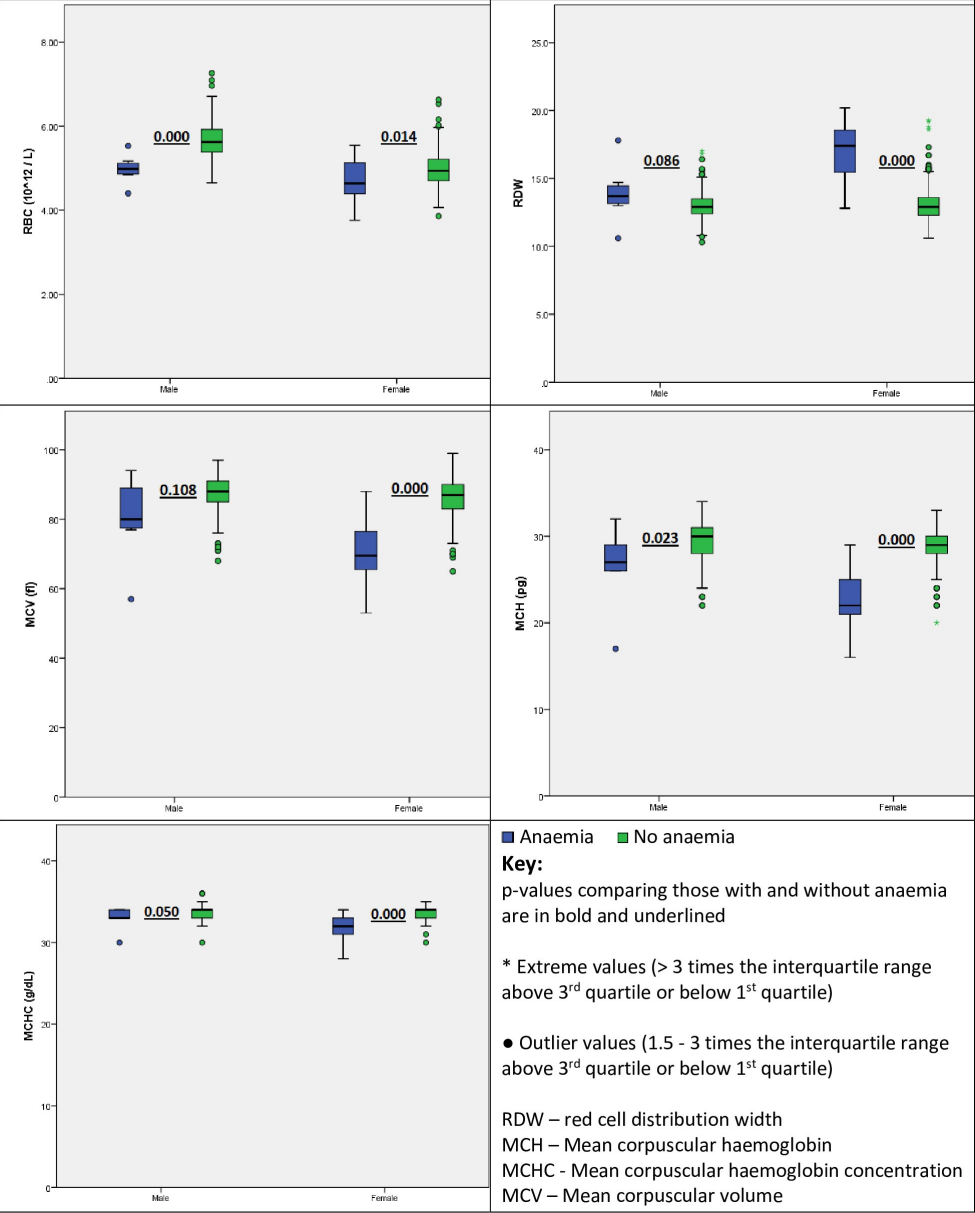
Red cell indices in participants with and without anaemia.

**Fig 2:**
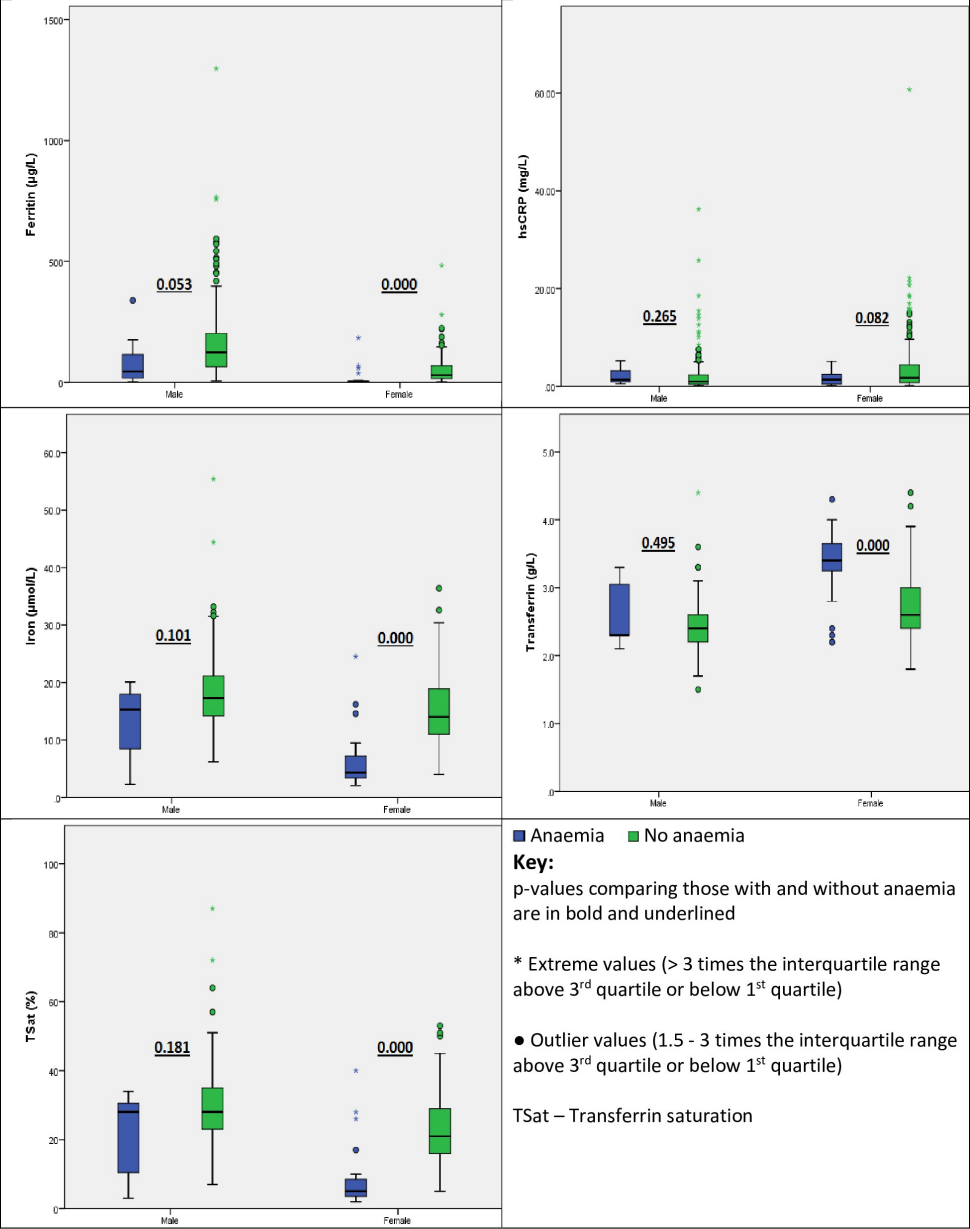
Distribution of iron status and inflammatory biomarkers in participants with and without anaemia.

Female participants with anaemia had statistically significant higher RDW and transferrin but lower RBC count, MCV, MCH, MCHC, ferritin, iron and TSat compared to those without anaemia as shown in Figs 1 and 2. A similar trend was observed in males however this did not reach statistical significance for RDW, MCV and markers of iron status. The difference in hsCRP was not statistically significantly different in those with and without anaemia as shown in Fig 2.

## Discussion

This estimated the burden of iron deficiency and determined the association between ferritin and anaemia in a healthy adult population in Kenya. The overall prevalence of iron deficiency using a ferritin cut-off of 15 μg/L and 30 μg/L was 15.3% and 29.9% respectively. A similar study carried out in South Africa by Dineo et al found a prevalence of 39.8% using a cut-off of 30 μg/L with black South Africans having the highest prevalence of 50.7% compared to 43.4% and 35.5% in the mixed ancestry and Caucasian participants respectively [15]. The prevalence of iron deficiency in our study population was much lower than what has been seen in South Africa possibly reflecting genetic and/or nutritional differences. In the South African study, the group with iron deficiency was predominantly women in the reproductive age group with a median age of 23 years, consumed less meat and had a lower proportion of people consuming vegetables daily compared to those who were iron replete [15]. Meat and green leafy vegetables are rich sources of haem and non-haem iron. The South African study excluded individuals with a CRP ≥ 10 mg/L with the assumption that this may have falsely increased ferritin which is an acute phase protein. However, the study didn’t report whether the excluded individuals had relatively higher ferritin levels.

The prevalence of anaemia in our population based on the reference interval cut-offs was quite low at 2.6% and 9.4% for males and females respectively with an overall prevalence of 5.9%. Using the WHO Hb cut-offs, anaemia prevalence reduced to 0.4% in males and overall prevalence to 4.7% which is quite low compared to the estimated global prevalence of 12.7% and 29.0% for males (15–60 years) and non-pregnant females (15–49 years) respectively [1,16]. Dineo et al reported an anaemia prevalence in South Africa of 2.9% and 18.3% for males and females respectively based on WHO cut-offs. Of note was that the overall anaemia prevalence in black South Africans was found to be 31.9% compared to 17.2% and 6.1% in the Mixed-ancestry and Caucasians respectively [15]. Majority of participants recruited in the Kenyan study were from Nairobi which is at an altitude of 1795 meters above sea level compared to Cape town which is close to sea level where majority of South African participants were recruited. Increase in altitude is associated with an increase in Hb due to a reduction in partial pressure of oxygen and increase in erythropoietin [17]. Indeed, Hb levels in Nairobi have been found to be much higher than in individuals living at sea level along the Kenyan coast [18].

Iron deficiency is the commonest cause of anaemia globally [19]. We made an assumption that the anaemia seen in our study participants was most likely due to iron deficiency and this was supported by the fact that those with anaemia had a higher RDW but lower RBC count, MCV, MCH and MCHC which is consistent with iron deficiency. Not surprisingly, this was predominantly seen in the female participants majority of whom were in the reproductive age and are generally at a higher risk of iron deficiency anaemia primarily due to menstrual blood loss. A similar pattern in RBC indices was seen when comparing those and without anaemia among male participants though for some of the parameters like RDW and MCV, this did not reach statistical significance possibly due to the fact that the number of males with anaemia was low. The prevalence of suspected iron deficiency and iron deficiency anaemia was very low in our study, possibly a reflection of the relatively good health of the recruited study participants. We explored the association between various ferritin cut-offs and anaemia presumed to be due to iron deficiency. Of the recommended ferritin-cut offs, 15 μg/L had the strongest association with predicting anaemia in both male and female participants. However, the positive predictive values was only 33% with a negative predictive value of 98% highlighting its usefulness in ruling out but not ruling in iron deficiency as a cause of anaemia. The less than impressive positive predictive value at a cut-off of 15 μg/L is not surprising given that we have previously published reference intervals for ferritin from this same population and found lower limits of 10 μg/L and 2 μg/L in males and females in the reproductive age respectively [12]. The WHO ferritin cut-off for diagnosing iron deficiency of 15 μg/L was derived from a random sample of 38-year-old women from Sweden recruited in 1968–1969. This cut-off was shown to best discriminate between iron-replete and iron-deficient women based on the absence of stainable iron in bone marrow smears but not the presence of anaemia [20]. In the absence of bone marrow examination and in light of the fact that iron deficiency is the commonest cause of anaemia, we evaluated the ferritin cut-offs based on their association with suspected iron deficiency anaemia. The WHO cut-off of 70 μg/L that is recommended in settings of inflammation would over diagnose iron deficiency in male adults in our setting while the cut-off of 15 μg/L would under diagnose the same in our population. Seventy-five female participants had ferritin levels below 15 μg/L but only 26.7% had anaemia indicating that the rest possibly had iron deficient erythropoiesis, a precursor to iron deficiency anaemia. Six male participants had a ferritin level below 15 μg/L but only 1 had a Hb below 13 g/dL which defines anaemia according to WHO. Reference intervals for ferritin are lower in females and as demonstrated in this study, ferritin levels were much lower n females hence cut-offs for diagnosing iron deficiency should be sex specific to avoid misclassification.

TSat has been proposed as an alternative biomarker to assess iron status in populations with chronic inflammation as it is less impacted by inflammation [5,6]. It is useful when assessing patients suspected to have functional iron deficiency especially in patients with anaemia but normal or increased ferritin levels. Based on our results, more than 91.7% of patients who had anaemia and ferritin less than 15 μg/L also had TSat levels < 20%. The positive predictive value of a TSat < 20% in predicting iron deficiency in patients with normal Hb was low (31.5%) hence its utility in assessing iron deficiency in a population similar to ours would be limited. We had expected that a larger proportion of participants with anaemia and normal or increased ferritin would have low TSat levels given that our population has low grade chronic inflammation attributed to a metabolic syndrome prevalence of 25.6% and a much higher CRP reference interval upper limit compared to a Caucasian population [9,12]. Of the individuals with ferritin < 70 μg/L, 45.4% (129 out of 284) had a TSat < 20% compared to 82.7% (67 out of 81) for those with a ferritin < 15 μg/L. The ferritin cut-off of 70 μg/L would therefore misclassify a higher proportion of individuals as having iron deficiency despite having normal TSat levels hence would not be appropriate in determining the presence of iron deficiency in the study population.

Our study is limited by the fact that our diagnosis of iron deficiency was not based on assessment of bone marrow iron stores but rather WHO ferritin cut-offs that have not been validated in an African population. It is possible that we may have misclassified some participants as having iron deficiency anaemia. However, the fact that participants with anaemia had RBC indices consistent with iron deficiency gives credence to our hypothesis that the anaemia in our participants was most likely secondary to iron deficiency. We did not provide iron supplementation and follow up for participants whose test results suggested that they had iron deficiency as this was an observational study. However, such participants were referred to their preferred primary healthcare giver with all the laboratory test results for further follow up and management. Monitoring the change in haemoglobin, RBC indices and markers of iron status for an appropriate response to iron supplementation would have served as an indirect indicator of iron deficiency in the absence of bone marrow assessment.

## Conclusion

Only the ferritin cut-off of 15 ug/L had an association with anaemia where it can be used for ruling out iron deficiency as the cause. Sex specific ferritin cut-offs for diagnosing iron deficiency in adults in sub-Saharan Africa need to be derived by comparing ferritin levels to stainable iron in bone marrow aspirates and trephines in order to ensure that we are using appropriate clinical decision limits. Majority of the participants in this study reside at a high altitude hence the findings can only be generalized to a population living in a similar environment given the impact of altitude on haemoglobin.
